# Ultrasound, Echocardiography, MRI, and Genetic Analysis of a Fetus with Congenital Diaphragmatic Hernia and Partial 11q Trisomy

**DOI:** 10.1155/2017/1471704

**Published:** 2017-03-02

**Authors:** Yolanda Fernández-Perea, Lutgardo García-Díaz, Javier Sánchez, Guillermo Antiñolo, Salud Borrego

**Affiliations:** ^1^Department of Genetics, Reproduction and Fetal Medicine, Institute of Biomedicine of Seville (IBIS), University Hospital Virgen del Rocío/CSIC/University of Seville, Seville, Spain; ^2^Centre for Biomedical Network Research on Rare Diseases (CIBERER), Seville, Spain

## Abstract

Congenital diaphragmatic hernia (CDH) is a serious birth defect with a significant mortality and morbidity. The current and constant progress in ultrasound techniques has led to the improvement of the prenatal diagnosis of this malformation. CDH is a developmental defect whose etiology is heterogeneous and takes place when the pleuroperitoneal folds and septum transversum fail to converge and fuse. Survival depends on the extent of pulmonary hypoplasia and the disease may be potentially worsened by the presence of added congenital defects. 40% of CDH cases are associated with at least one additional anomaly. The ultrasound diagnosis is established with essential signs: loss of uniform echogenicity of lungs and marked mediastinal shift. We report the case of a fetus with isolated CDH diagnosed at 21 weeks of gestation by ultrasound and confirmed by RMI, whose genetic analysis of amniotic fluid cells identified a de novo partial trisomy of the long arm of chromosome 11. Different genetic causes have been associated with CDH. Moreover, it is expectable that the use of new techniques for prenatal diagnosis will reveal novel CNVs associated with CDH and will help us to estimate the recurrence risk for this defect as well as for other associated anomalies.

## 1. Introduction

Congenital diaphragmatic hernia (CDH) is a serious birth defect that, despite advances in neonatal care, leads to a significant mortality and morbidity during the neonatal period [1, 2]. CDH occurs in 1 in 3000 live births and differential diagnosis includes cystic adenomatous malformation, teratoma, bronchogenic cyst, and extralobar pulmonary sequestration [3]. Technical improvements in early ultrasound examinations have increased the rate of prenatal diagnosis of different congenital anomalies including CDH. A high percentage of CDH is identified during prenatal ultrasound, when the liver and/or the intestines are presented in the chest together with a malpositioned heart. CDH impairs normal lung development, which implies pulmonary hypoplasia determining the fetus outcome [4]. The size of CDH is important in order to assess the risk of neonatal morbidity and mortality. Currently, prenatal MRI studies have been used in the differential diagnosis of echogenic, cystic intrathoracic masses [5]. Fetal echocardiography is essential since cardiac defects are the most common abnormality associated with CDH and it determines a possible early surgery [3]. Fetuses presenting with other malformations, chromosome abnormalities, and/or syndromes together with or including CDH show a lower survival rate than those with isolated CDH [4].

The wide variety of genetic abnormalities associated with either isolated or syndromic CDH reflects the heterogeneity of this malformation. Chromosomal abnormalities including aneuploidies, genomic deletions, and duplication are frequently identified in fetuses with nonisolated CDH. Mutations in CDH forms with autosomal recessive, autosomal dominant, or X-linked patterns of inheritance have been identified [6]. However, the high percentage of CDH cases of unknown origin suggests the existence of other nongenetic causes [6].

Here, we report the case of a fetus with isolated CDH diagnosed at 21 weeks of gestation. Ultrasound examination, echocardiography, MRI, and genetic analysis were performed. We discuss the usefulness of these techniques in the diagnosis of a fetus with either isolated or nonisolated CDH.

## 2. Case Presentation

A healthy 37-year-old woman was examined at 16 weeks of gestation because of a positive first-trimester screening. There was neither consanguinity nor familial history of genetic disease or miscarriage. The couple previously had a healthy child. Ultrasound scan showed normal fetal biometry, with a biparietal diameter of 36.9 mm, frontooccipital diameter of 45.8 mm, head circumference of 129.8 mm, abdominal perimeter of 104.9 mm, and femur length of 21.4 mm, as well as an estimated fetal weight of 159 g. The patient refused amniocentesis for cytogenetic analysis.

**Figure 1:**
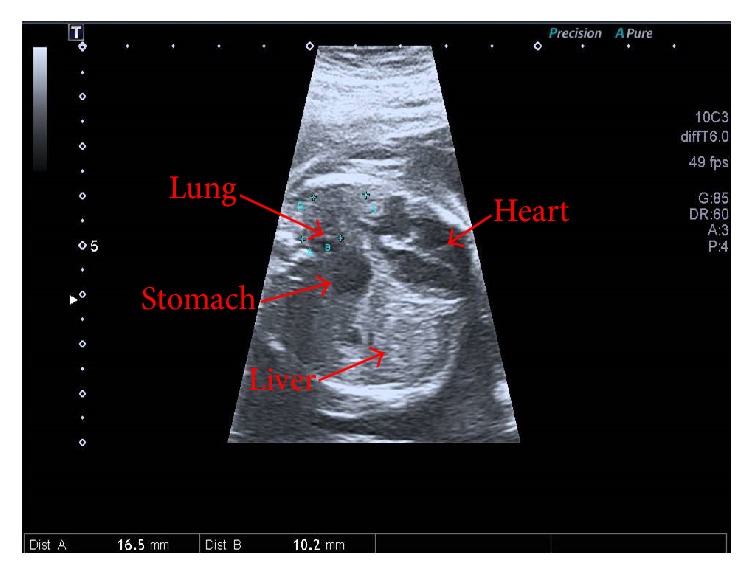
Ultrasound image. Ultrasound image in which we observe stomach and liver in the chest cavity next to four-chamber view of the heart and left lung's volume reduced.

Subsequently, ultrasound scan at 20 + 4 weeks of gestation showed a CDH on the left side (Figure 1). The fetal biometry showed a biparietal diameter of 48.8 mm, frontooccipital diameter of 58.8 mm, head circumference of 172.9 mm, abdominal perimeter of 137.7 mm, femur length of 37.2 mm, and an estimated fetal weight of 353 g. A detailed ultrasound examination showed a full-filled stomach within the chest cavity, mediastinum shift, and dextrocardia. Given those findings, the patient decided to accept amniocentesis for genetic analysis.

### 2.1. MRI Exam

MRI was performed at 21 + 5 weeks of gestation. MRI showed wide left diaphragmatic herniation that included the left hepatic lobe, the stomach, and the spleen as well as the colon and the bowel loops. This extensive hernia caused cardiomediastinal shift and bilateral lung hypoplasia. Volumetric right lung was 1.22 cc, while volumetric left lung was not possible to determine because of extensive lung hypoplasia.

### 2.2. Cytogenetic and FISH Analysis

Two independent amniotic fluid cells cultures were set up. We analyzed 20 metaphases with chromosomal standard GTG banding techniques with a minimum resolution level of 550 bands.

Amniotic fluid cell chromosome preparations hybridized with the LSI* MLL* Dual Color Break Apart Rearrangement Probe (11q23)kit [Vysis, Downers Grove, IL] were analyzed. This kit consists in a combination of two probes that hybridize in the* MLL* locus: a proximal LSI* MLL* Flanking Probe (SpectrumGreen) and a distal* MLL* Flanking Probe (SpectrumRed). In addition, FISH analysis with the subtelomeric probe* D11S1037* (11qter) [Vysis, Downers Grove, IL] was performed on slides with the amniotic fluid cells according to supplier protocols.

**Figure 2:**
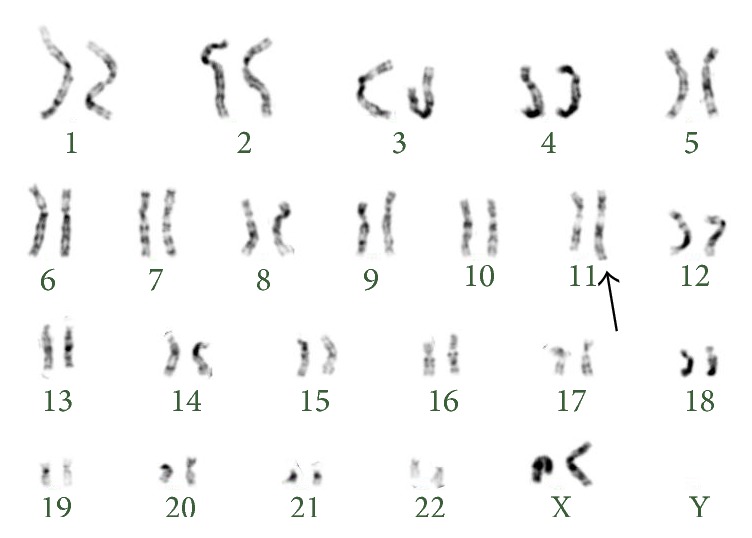
Karyotype of amniotic fluid cells showing additional material in the long arm of chromosome 11 (black arrow).

**Figure 3:**
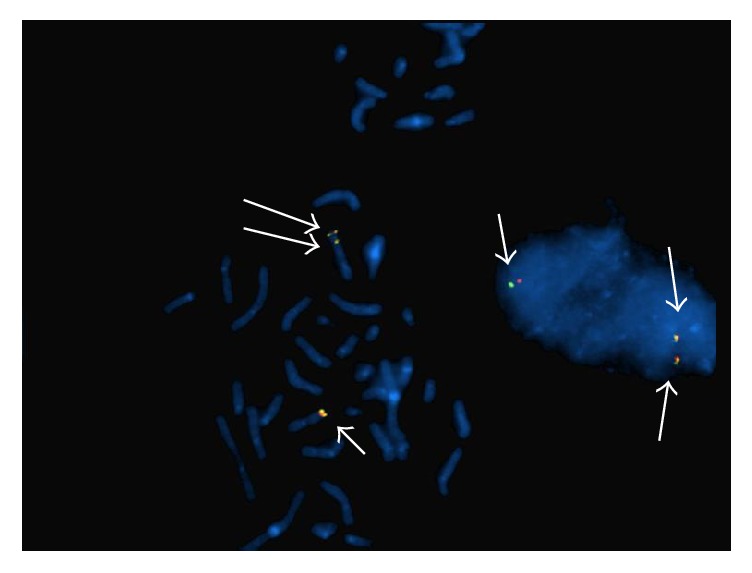
FISH analysis with MLL probes. Partial metaphase with MLL probes showed two signals in the 11q chromosome. Interphase nuclei showed three signals for MLL probes. White arrows show MLL hybridization pattern.

Karyotype revealed additional material in 11q (Figure 2). FISH analysis allowed us to identify an extra signal for* MLL* probes in interphase nuclei. Analysis in the metaphase cells showed an extra signal in the long arm of chromosome 11 (Figure 3). Only cells showing signals for both the proximal LSI MLL Flanking Probe (SpectrumGreen) and the distal MLL Flanking Probe (SpectrumOrange) were scored. FISH analysis with the subtelomeric probe showed a normal FISH pattern. These findings were compatible with tandem duplication of 11q chromosome material. Karyotype formula was established as 46,XX,dup(11)(q13.5q24).ish dup(11)(MLL++,D11S1037+).

A cytogenetic study was performed for the parents in order to discard any familial rearrangement. Parental karyotypes resulted to be normal.

Taking together the cytogenetic results, together with the ultrasound and MRI findings, the parents decided to terminate the pregnancy at 23 + 4 weeks of gestation.

Autopsy confirmed a female fetus presenting with an extensive left side congenital diaphragmatic hernia with left lung hypoplasia.

## 3. Discussion

CDH is most often diagnosed during morphological ultrasound examination performed at the 18th–22nd week of pregnancy. During this stage, ultrasound shows the diaphragm as a fine hypoechogenic structure between the thorax and the abdomen. Experience of ultrasound examiners, gestational age, fetus position, side of the lesion, extension of the alteration, and the existence of additional malformations are aspects that influence the ability to identify prenatally this malformation [7]. Left side CDHs are easier to be identified during ultrasound examination because they affect the stomach, small bowel, and colon. Extensive hernias are associated with cardiomediastinal shift and bilateral lung hypoplasia and consequently with a poor prognosis [4, 8]. Bilateral CDHs are very uncommon and they are associated with a fatal outcome.

The severity of lung hypoplasia is determined by the lung-to-head ratio (LHR). LHR is the ratio between the lung area contralateral to the hernia obtained at the level of a four-chamber view of the fetal heart in cross-sectional images and the head circumference of the fetus. LHR > 1.4 is associated with a high survival rate, between 1 and 1.4 with a moderate survival rate, and <1 with a very low survival rate. In this case, the fetus showed LHR < 1. The observed-to-expected lung-to-head ratio expressed as percentage (O/E LHR) was 24%, associated as well with a poor prognosis.

MRI has been applied for the prenatal diagnosis of this pathology [9]. MRI allows obtaining a high resolution level of the lesion and of the fetal organs and is independent of the fetus position, the side of the lesion, and gestational age. Total fetal lung volume and right and left lung volumes are determined and compared with normal lung volumes for a specific time of gestation. These values can be obtained by either ultrasound or MRI. Volumetric lung measurements are a predictive postnatal outcome factor. In our particular case, since the two main predictions of postnatal outcome rates showed a poor prognosis for the fetus, we offered again a late amniocentesis.

Many genetic causes have been identified during prenatal diagnosis of fetal CDH. The most prevalent aneuploidies include trisomy 18 and trisomy 13 [2], as well as sex chromosomes aneuploidies [10–12]. Complete autosomal trisomy in fetal CDH is always associated with additional prenatal morphological abnormalities. Large chromosome deletions and duplication have been also associated with CDH. The most frequent deletion syndrome associated with CDH is the deletion of 8p23.1. This syndrome presents with Central Nervous System (CNS) anomalies, characteristic facies, mental retardation, and autism. This region includes the* GATA4* gene implicated in the development of the diaphragm [13]. Other deletion syndromes associated with CDH and heterogeneous malformations included deletions of 15q26.1 [14], 1q41 [15], or 8q23.1 [16].

CDH has been associated as well with partial 11q23.2 duplication too. However, those partial 11q trisomies were associated with other chromosome anomalies resulting from unbalanced translocations, so that those patients showed additionally a partial monosomy in other chromosomes [17, 18]. In the case present here, parental cytogenetic analyses were normal and no other chromosome region was involved. Therefore, we conclude that the duplication 11q observed during prenatal diagnosis was a de novo event. In this situation, recurrence risk might be very low.

Different genes located at 11q have been proposed to play a role in the development of CDH [19]. The* ROBO4 *gene is expressed in the endothelial cells and it is essential for coordinated symmetric and directed sprouting of intersomitic vessels. The* CDON* gene is implicated in differentiation and transformation of cells in the skeletal muscle lineage. Therefore, the additional copies of these genes may contribute to CDH in patients with duplication of 11q24.2.

In summary, CDH is a heterogeneous entity. Prenatal diagnosis by ultrasound and MRI will help to evaluate the extensiveness of the malformation and to determine the prognosis and the treatment. It is essential that the genetic specialist should be present when providing the genetic information to the affected family. In fetuses with CDH, a genetic analysis by array CGH combined with a cytogenetic analysis will probably allow detecting the genetic cause of the malformation and hopefully predicting the prognosis.
